# Adaptation of Oxford Nanopore technology for hepatitis C whole genome sequencing and identification of within-host viral variants

**DOI:** 10.1186/s12864-021-07460-1

**Published:** 2021-03-02

**Authors:** Nasir Riaz, Preston Leung, Kirston Barton, Martin A. Smith, Shaun Carswell, Rowena Bull, Andrew R. Lloyd, Chaturaka Rodrigo

**Affiliations:** 1 Kirby Institute, UNSW Sydney, Sydney, NSW 2052 Australia; 2 Department of Microbiology, Hazara University, KPK, Maneshra, 21120 Pakistan; 3 Kinghorn Centre for Clinical Genomics, Garvan Institute of Medical Research, Sydney, Australia; 4 Department of Pathology, School of Medical Sciences, UNSW Sydney, Sydney, NSW 2052 Australia

**Keywords:** Hepatitis C virus, Third generation sequencing, Nano-Q, Haplotypes, Oxford Nanopore technology

## Abstract

**Background:**

Hepatitis C (HCV) and many other RNA viruses exist as rapidly mutating quasi-species populations in a single infected host. High throughput characterization of full genome, within-host variants is still not possible despite advances in next generation sequencing. This limitation constrains viral genomic studies that depend on accurate identification of hemi-genome or whole genome, within-host variants, especially those occurring at low frequencies. With the advent of third generation long read sequencing technologies, including Oxford Nanopore Technology (ONT) and PacBio platforms, this problem is potentially surmountable. ONT is particularly attractive in this regard due to the portable nature of the MinION sequencer, which makes real-time sequencing in remote and resource-limited locations possible. However, this technology (termed here ‘nanopore sequencing’) has a comparatively high technical error rate. The present study aimed to assess the utility, accuracy and cost-effectiveness of nanopore sequencing for HCV genomes. We also introduce a new bioinformatics tool (Nano-Q) to differentiate within-host variants from nanopore sequencing.

**Results:**

The Nanopore platform, when the coverage exceeded 300 reads, generated comparable consensus sequences to Illumina sequencing. Using HCV Envelope plasmids (~ 1800 nt) mixed in known proportions, the capacity of nanopore sequencing to reliably identify variants with an abundance as low as 0.1% was demonstrated, provided the autologous reference sequence was available to identify the matching reads. Successful pooling and nanopore sequencing of 52 samples from patients with HCV infection demonstrated its cost effectiveness (AUD$ 43 per sample with nanopore sequencing versus $100 with paired-end short read technology). The Nano-Q tool successfully separated between-host sequences, including those from the same subtype, by bulk sorting and phylogenetic clustering without an autologous reference sequence (using only a subtype-specific generic reference). The pipeline also identified within-host viral variants and their abundance when the parameters were appropriately adjusted.

**Conclusion:**

Cost effective HCV whole genome sequencing and within-host variant identification without haplotype reconstruction are potential advantages of nanopore sequencing.

**Supplementary Information:**

The online version contains supplementary material available at 10.1186/s12864-021-07460-1.

## Background

RNA viruses such as dengue, hepatitis C (HCV), zika, and influenza are pathogens responsible for a significant proportion of global infectious diseases in both high- and low-middle income countries [1]. Each of these infections have varied disease phenotypes in humans (e.g. haemorrhagic fever versus simple fever in dengue, or chronic infection versus spontaneous clearance in HCV) which may be associated with viral genomic characteristics [2]. Better methods for high throughput viral genome sequencing are essential to design predictive, preventative (using phylogenetics to detect and control emerging clusters of infection) and curative strategies against RNA viral infections. Given the lack of proof-reading capacity and the high replication rate, any host infected by a single RNA virus has multiple, heterogenous, yet related viral variants [3]. These within-host viral variants evolve over time in response to host selection pressures either by generating escape mutations against natural host immunity, or drug-resistant variants in individuals treated with antiviral drugs. Some of these escape or resistant mutations may have a fitness cost which impairs the replication capacity, which the virus seeks to balance (to reduce the fitness cost) by selecting variants with co-occurring mutations on the same genome [4]. Improved understanding of the influence of viral genomics on disease phenotypes requires a detailed examination of the mutational landscape of within-host variants in RNA viruses.

Until a decade ago, it was largely impossible to characterise within-host viral variants. This could be done with single genome amplification in combination with Sanger (first generation) sequencing, but this approach was expensive, laborious and unsuitable for high throughput sample processing. With second generation sequencing technologies (also known as next generation sequencing - NGS), mutations occurring at a frequency as low as 0.1% in the viral population can be reliably identified [5]. These technologies are currently offered on multiple commercial platforms with the most popular being the paired-end short read sequencing offered by Illumina™. RNA genomes are relatively small (~ 5000–35,000 nt), but none of the first- or second-generation sequencing platforms can generate reads of full genome length. It is possible with NGS to estimate the distribution of within-host viral variants bioinformatically by performing haplotype reconstruction, in which short reads that are likely to originate from the same variant are ‘stitched together’ and then extended to form an estimated viral variant [6, 7]. Currently, there are multiple algorithms for viral haplotype reconstruction, but these do not have good concordance with each other for the same dataset [8]. Since there is no gold standard, it is time consuming and difficult to determine the best haplotype reconstruction tool for a specific sequence data set. As haplotype reconstruction algorithms estimate whether individual reads belong to the same viral variant based on shared mutations within overlapping short reads, they are biased by errors in the algorithm as well as by technical errors in the sequencing technology.

Third generation sequencing technologies are now available commercially and generate long reads far exceeding the average length of RNA virus genomes. They are offered on two main platforms: Pacific Biosciences (currently under a purchase agreement with Illumina) and Oxford Nanopore Technology (ONT). These methods offer the first opportunity to sequence whole viral genomes as single reads, thereby potentially enabling detailed and reliable characterisation of within-host viral variants. Of the two commercial platforms, ONT has the added advantage of using a portable sequencer (MinION) that can be linked to a standard computer enabling real-time sequencing in the field or in remote locations without the need for a sophisticated laboratory [9]. However, ONT reads (henceforth referred to as nanopore reads/sequences) have a high error rate (10% vs. 0.1%) compared to paired-end short reads generated on the Illumina platform (henceforth referred to as short read technology), which limits the reliability and usefulness of its long reads. If optimized, this technology may solve the longest standing problem in RNA virus genomics, that is accurate and cost-effective sequencing of within-host viral variants. The cost of sequencing can be further reduced by tagging the PCR products of a sample with a synthetic oligo-nucleotide segment (a barcode), which allows pooling of multiple samples (multiplexing) prior to sequencing and de-multiplexing (separation of reads by barcodes) afterwards.

This paper describes an assessment of the utility of nanopore sequencing, in terms of coverage, accuracy and cost, for near full-length HCV genome sequencing using reverse transcribed cDNA amplicons as template. In addition, a novel bioinformatics pipeline was designed for identification of within-host viral variants using nanopore data.

## Results

### Nanopore technology generates comparable consensus sequences to short read (Illumina) technology

**Fig. 1:**
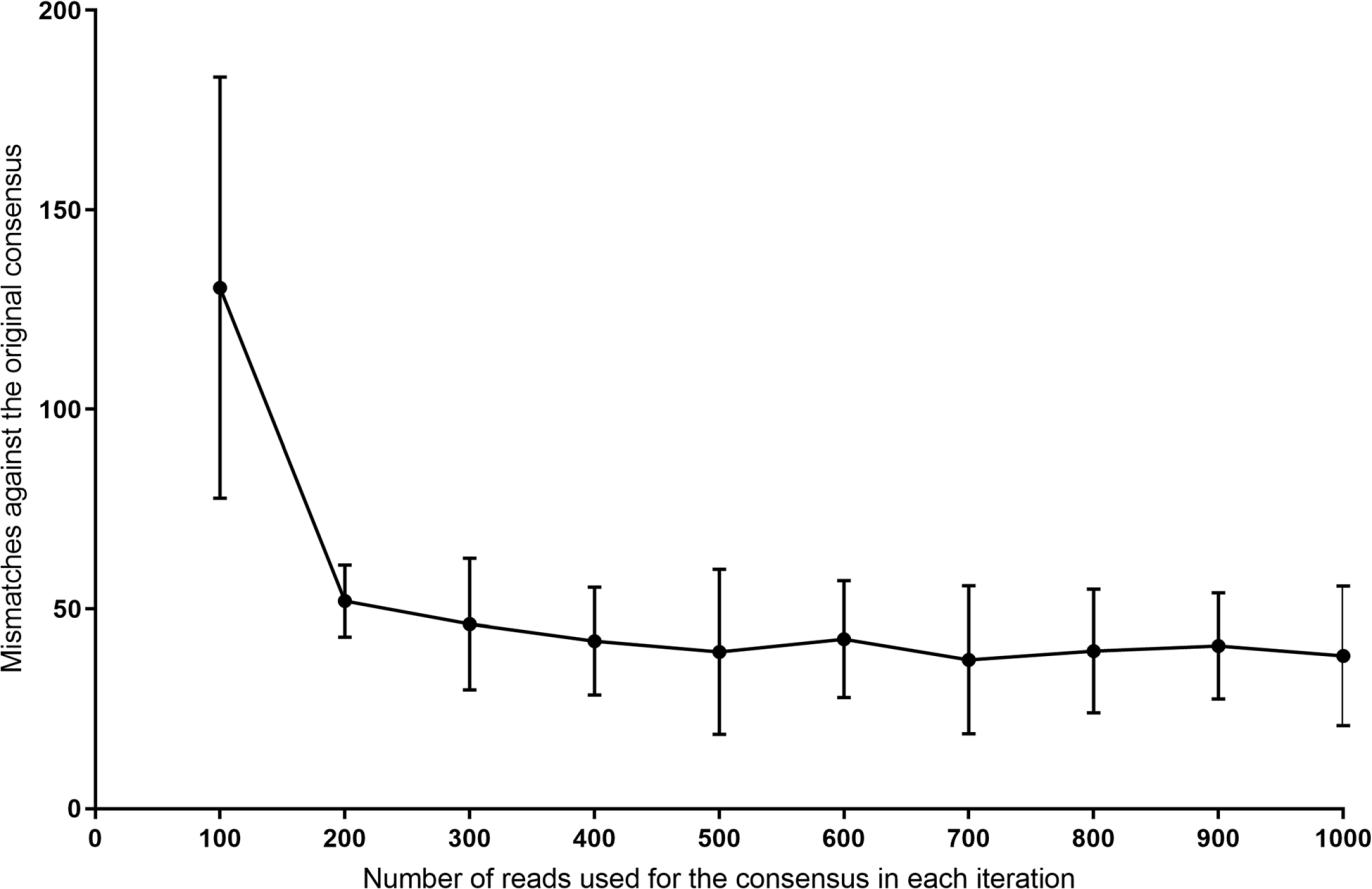
The minimum number of nanopore reads required to generate an accurate consensus sequence. Four HCV full length amplicons were sequenced with both Illumina (Miseq) and nanopore platforms. Consensus sequences made from randomly picked nanopore reads (in multiples of 100, each read > 8500 nt) were compared against the consensus sequence made from the entire volume of Illumina reads which had an average coverage of 17,000 nt per position (used here as the gold standard). Each data point demonstrates mean pairwise mismatches and standard deviation. The accuracy does not improve further beyond 300 nanopore reads

To test the ability of nanopore technology to generate an accurate consensus sequence, five HCV subtype 1a amplicons (each originating from a single patient with HCV infection) were simultaneously sequenced with nanopore and short read sequencing platforms. The consensus sequences from each alignment were compared (Fig. 1). The pairwise mismatches between the short-read consensus and the nanopore read consensus was on average 0.37 per 1000 bases (standard deviation; SD ± 17.74). To determine the minimum number of nanopore reads required to make an accurate consensus (assuming the short read sequences were gold standard), sequences meeting a minimum length cut-off (> 8.5 kb) were randomly drawn from the total pool in multiples of 100 to generate a consensus sequence, which was then compared with the consensus generated from short reads (Fig. 1). After the nanopore read coverage exceeded 300, the accuracy of the consensus did not improve further (beyond 98–99% similarity).

### Nanopore sequencing can identify low frequency variants

Two experiments were conducted to determine if low frequency variants could be detected. Experiment 1 (Exp1) mixed one major HCV sequence insert of a plasmid (at relative frequencies of 84–93% in abundance within the surrogate quasi-species) with 4 other plasmids, each carrying a different HCV insert (< 5% abundance). The insert size was approximately 1800 nt, comprising the Envelope region of HCV open reading frame. The pairwise differences between the inserts were > 15% for different subtypes, and between 5 and 15% within the same subtype. Two plasmids had inserts isolated from the same patient at different time points of the infection with a < 5% pairwise difference. Five different plasmid mixes were made as above, and tagged with one nanopore barcode per mix (by ligation). The lowest frequency of a plasmid in any one of these mixes was 0.1%.

**Fig. 2:**
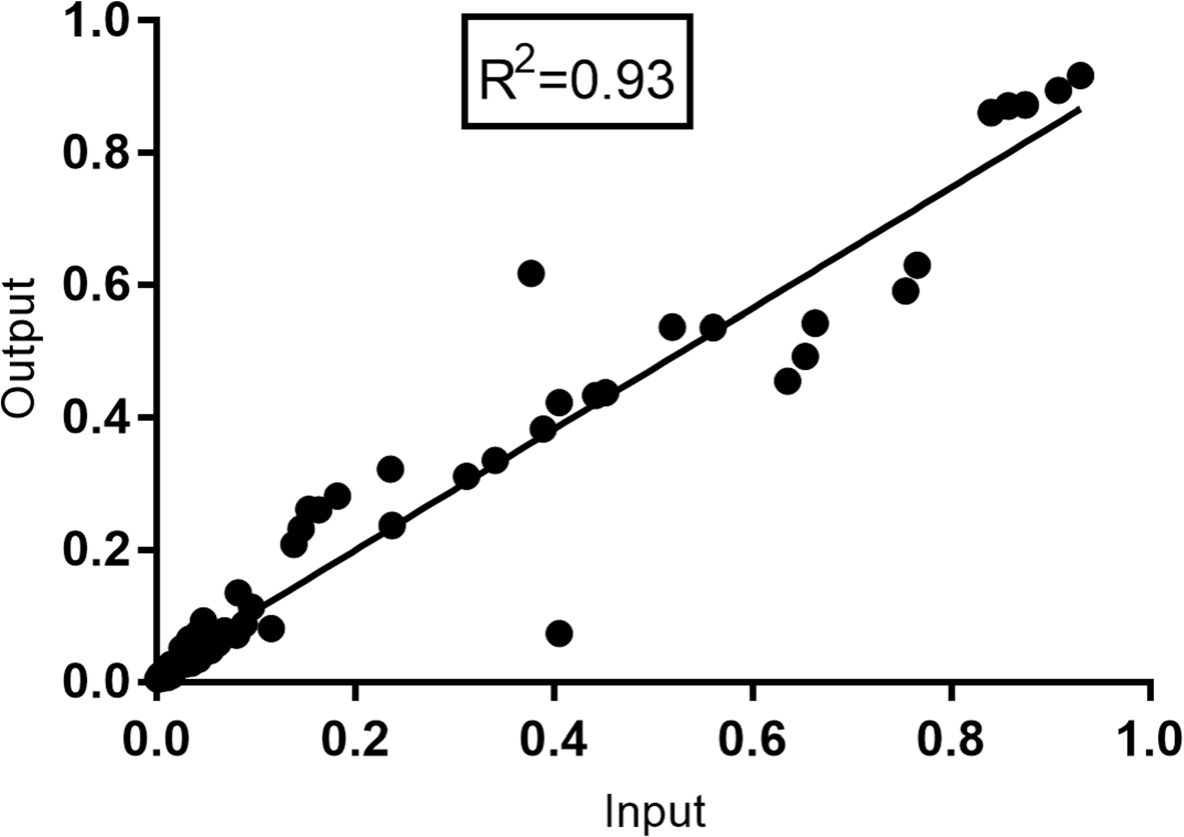
Accuracy of nanopore sequence output in reproducing high and low frequency variants in a mix of sequences. Plasmids with Hepatitis C virus E1E2 inserts (1800 nt) were mixed in different proportions (0.1–93%) with 5 plasmids per mix and approximately 15 such mixes. Each mix were tagged with the same nanopore barcode and sequenced on the same flow cell. The original proportions of each insert could be reproduced post-sequencing even when the input frequency was as low as 0.1%. The original plasmid insert sequence was used as a reference to identify corresponding nanopore reads. X axis- input plasmid frequency calculated as a % based on concentration, Y-axis output frequency calculated as the number of nanopore reads per HCV insert as a % of the total nanopore reads per mix

For experiment 2 (Exp2) the number of mixes was increased to 10 with a wider representation of plasmid frequencies between 0.6–76% across all mixes (See Supplementary Methods). After nanopore sequencing, the coverage per insert in each mix ranged from 10^4^ to 10^5^ reads. The number of pairwise mismatches between the reconstructed HCV sequence and the sequence of the original plasmid insert was on average 2.11 per 1000 nt (SD ± 2.41) across all inserts and mixes. The comparison of relative frequencies between the input and the nanopore output (actual versus reconstruction from nanopore sequencing) from both experiments showed that nanopore sequencing accurately reproduced the original plasmid frequencies across a broad range of abundance from 0.1 to 93% (Fig. 2).

### Nanopore sequencing is cost effective for high throughput HCV sequencing

To assess cost effectiveness, 52 HCV patient samples were sequenced in a single flow cell (with PCR-based barcoding followed by sequencing on the GridION platform). These samples included 6 different HCV subtypes; 1a, 1b, 2a, 3a, 4a and 6I (*n* = 30, 1, 5, 14, 1, and 1 respectively). Reads for all samples were recovered after de-multiplexing. The nanopore sequencing run produced on average 5141 reads per sample (range 224–18,893) with a total output of 1.27 million reads (6.82Gbp total yield) during a run time of 47 h. The mean quality per base call was Q8.7 with a median read length of 9.1 kb. The median pairwise mismatches between the Illumina consensus and the nanopore consensus for the near full-length HCV genome (approximately 9000 kb) was 7 (IQR: 5–13, Fig. 3). Nanopore sequencing was significantly cheaper with a per sample cost of AUD$ 43 in comparison to AUD$ 100 for Illumina sequencing (estimates based on reagent costs in May 2019 in Australia). The cost comparison includes the cost of library preparation, in addition to that of sequencing.

**Fig. 3:**
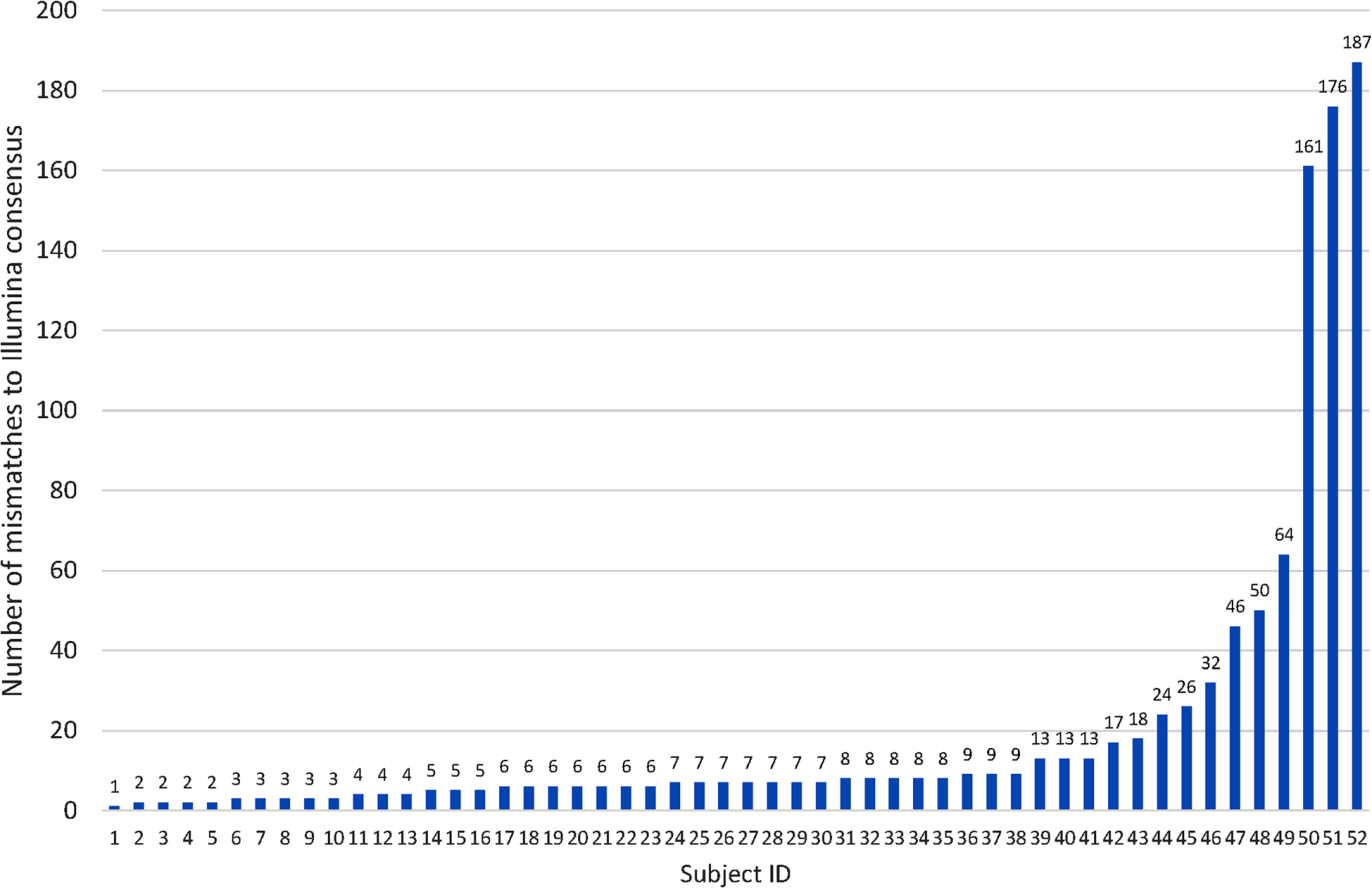
Accuracy of pooling multiple samples with PCR based barcoding for nanopore sequencing on the same flow cell. 52 full-length HCV amplicons isolated from different patients were sequenced concurrently on Nanopore (with PCR based barcoding) and Illumina platforms and pairwise mismatches were compared across consensus sequences. For samples with a high number of mismatches, either nanopore or Illumina sequence did not have an adequate coverage in some segments of the genome (adequate coverage was defined as > 300 reads for nanopore and > 100 reads for Illumina)

### Differentiation of between host read clusters without autologous references

**Fig. 4:**
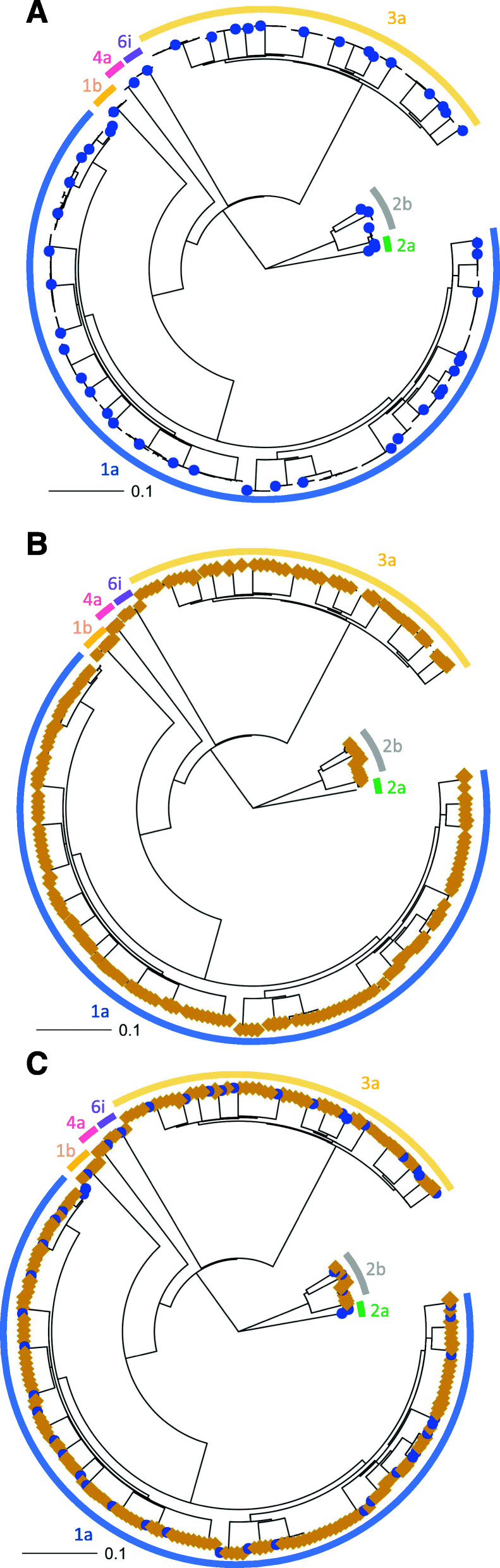
Identification of within host variants with Nano-Q tool. The within host variants identified by Nano-Q tool are represented as brown squares while consensus sequences generated from Illumina sequences are represented by blue dots. Clades from different HCV subtypes are named on the figure (Neighbour joining tree, bootstrap support > 90%). Panel **a**: Illumina consensus sequences only (Nanopore variants hidden), Panel **b**: Nanopore sequenced within host variants (Illumina consensus hidden), Panel **c**: All sequences shown

The entire output from the 52-sample nanopore run was used to test the Nano-Q tool, which is a new bioinformatic tool (Nano-Q) designed by the authors to separate within-host viral variants using nanopore sequencing data. When a single subtype 1a reference sequence was provided to the pipeline with all reads as the input (i.e without subject-specific de-multiplexing), the Nano-Q tool successfully selected all of the subtype 1a reads and accurately arranged them into accurate subject-specific clusters by comparing Hamming distances using a hierarchical clustering approach. The accuracy of this step was confirmed by combining consensus sequences generated from paired end short read sequencing (Illumina) with nanopore sequenced variants in the same phylogenetic tree (Fig. 4, Supplementary files 7 and 8). Each of the Illumina-generated consensus reads clustered with the respective nanopore-generated variants, and there was no mixing of variants between clusters. Similar results were obtained for other subtypes by provision of an appropriate subtype-specific sequence as the reference. These data show the capacity of Nano-Q to separate subject-specific sequences from a complex mix of sequences from multiple subjects even without barcoding.

### Differentiation of within-host viral variants

**Fig. 5:**
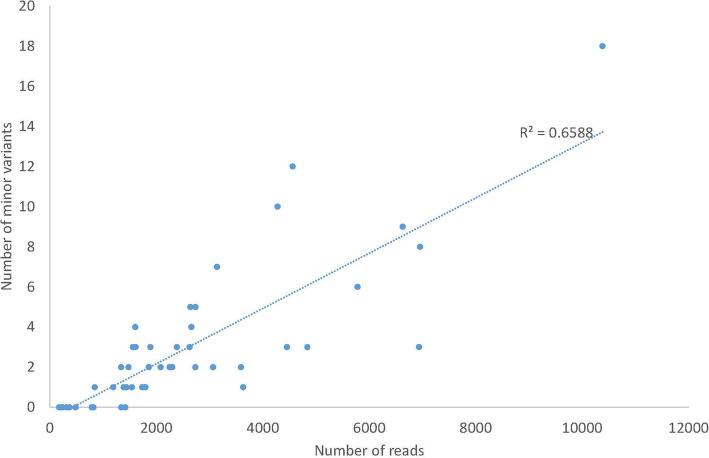
Relationship between the number of low frequency variants (< 5% abundance) and the number of input reads for the Nano-Q tool. If more reads are eligible to enter the full Nano-Q pipeline (after the initial steps of cleaning and size selection), more low frequency variants are detected. There was no saturation in the number of variants within range of eligible reads examined. However, as shown in text, detecting more low frequency variants did not cause significant changes in the frequency of major variants

When demultiplexed, subject-specific sequences were used as the input to the Nano-Q tool using the recommended parameters (−ht: 400, −mc: 20, see Methods for details), a total of 1–22 (median: 6, IQR: 4–9) within-host variants were identified per subject across the 48 subjects (in 4 subjects, the eligible read number after cleaning step were too few for a meaningful interpretation). Manual inspection of these variants demonstrated SNPs (not ambiguities, insertions or deletions) with a median pairwise mismatch of 6 (IQR of 4–14.5) per 8919 bases (as a percentage, median: 0.07%, IQR: 0.05–0.16%) across variants from a single host. A sensitivity analysis was performed by varying several parameters of the pipeline [e.g. reducing the length of eligible reads (−l) from 9000 to 2000; reducing the minimum cluster size (−mc) from 30 to 20] and these approaches recognized an additional 1–3 low frequency variants, but had limited impact on the frequencies of major variants (> 5% abundance). The total number of low frequency variants detected was also dependent on the number of eligible reads remaining after the initial cleaning step (Fig. 5).

## Discussion

Nanopore sequencing can be successfully and cost-effectively employed for full genome sequencing of HCV. This platform is comparable in accuracy to short read (Illumina) sequencing to generate a viral consensus sequence for each subject, provided the minimum coverage exceeds 300 reads per nucleotide position. It also reliably differentiated low frequency variants within in silico HCV plasmid sequence mixes, when such variants had an abundance as low as 0.1%, provided that an autologous reference sequence was available. The coverage offered by ONT GridION technology makes it possible to combine up to 96 samples in a single flow cell while meeting the cut-offs above for accuracy, thus markedly reducing the cost of sequencing. The Nano-Q bioinformatics tool developed by the authors accurately separated nanopore read clusters originating from different subjects using a single, subtype-specific, non-autologous reference. Nano-Q was also able to identify within-host variants without an autologous reference sequence.

The ONT platform is becoming increasingly popular given its portability and ease of use without a large capital investment [10, 11]. The capacity to generate long reads provided by the ONT platform also enables sequencing of whole RNA viral genomes which are typically in the range of 10–30 Kb. Full genomes are not essential for the diagnosis of viral infections, but do offer substantial advantages for molecular epidemiological investigations, including phylogenetics, as well as studies of within-host viral epistasis [2, 12, 13]. Even for diagnostic purposes, given the low cost and limited expertise required, nanopore sequencing may offer a cheap and affordable alternative. As sequencing becomes cheaper for developing countries, the global bias in the geographical origin of public database sequences may disappear for neglected tropical infections, thereby enabling targeted research for heavily impacted low-income countries.

Prior to widespread roll-out of nanopore technology for RNA virus genomic studies, it is important to benchmark its accuracy against current state-of-art sequencing alternatives. The authors have previously studied the utility of different NGS platforms for HCV sequencing to document the strengths and limitations of each method for RNA virus sequencing [14, 15]. For example, the 454 pyrosequencing platform offers longer reads than paired-end short read (Illumina) technology, but has reduced accuracy in differentiation of single nucleotide polymorphisms (SNPs) and is prone to multiple spurious indels within a read alignment. In contrast, Illumina technology offers better quality alignments and accuracy in characterization of SNPs, but the short-read length is a barrier to reliable reconstruction of within-host viral variants (haplotypes). Single molecule real-time sequencing offered by Pacific Biosciences (PacBio) offers long reads exceeding the size of many RNA viral genomes but the sequencers are bulky, require sophisticated laboratory facilities, and at the moment are not very cost effective for high throughput sequencing [12, 14–16]. Nanopore sequences are longer, often exceeding the average length of an RNA virus genome, thus enabling whole genome sequencing. However, the technical error rate in base calling in nanopore sequencing is much higher when compared to paired-end short read technology (10% vs < 1%) [5]. This error rate continues to improve as new pore versions are introduced by the parent company (from so-called R6 to the currently used R9.5). In addition, there are several post-sequencing computational methods to further reduce the error rate [17]. However, if such errors are randomly distributed, then the consensus of relatively few reads (i.e coverage > 10) should be sufficient for an accurate consensus as random errors are not consistent across reads. Unfortunately, the distribution of errors are not random but are preferentially located at homo-polymeric regions, as shown by others previously [17, 18], and hence the coverage needs to be much larger to produce an accurate consensus as shown in this study (in the range of 200–300 reads). The extensive coverage obtained for each sample in the analysis presented here exceeded this coverage threshold even when more than 50 samples were pooled in a single flow cell.

Experiments with plasmid mixes documented the ability of nanopore sequencing to reproduce the original sequences in correct proportions down to a frequency of occurrence as low as 0.1%, when the reference sequence identified the matching reads from the total pool. This cut off may even be less than 0.1% as this was the lowest plasmid abundance included in the experiments reported here. The cut-off also depends on the yield of reads in the length of interest, which in turn is dependent on the number of samples pooled, input DNA amount per sample, and the total run time.

Nanopore sequencing is cost effective compared to other alternatives currently on the market and this margin of cost-saving may improve as more samples are pooled. If the aim is consensus level viral sequence analysis, then nanopore sequencing has comparable accuracy to the current state-of-art Illumina sequencing (which also allows pooling of multiple samples with barcoding). Extrapolating the results reported here for 52 samples, it is anticipated that even if the maximum possible sample numbers (*n* = 96) were to be pooled, it would still generate an adequate coverage per sample while lowering the sequencing cost to around AUD$ 24 per sample. However, if the aim is to identify the frequency of SNPs in an alignment of sequences, given the low quality score of individual base calls (on average Q7 with nanopore sequencing vs. Q30–40 with paired end short read technology), nanopore sequencing is currently not recommended.

The number of samples that can be pooled in a single run also depends on the barcoding method. Barcoding by ligation of oligonucleotides to the viral DNA is comparatively costly, tedious and only allows a maximum of 24 samples with commercial barcodes (technically, there is no barrier to pool more samples with custom made barcodes). By contrast, PCR-based barcoding is cheaper and faster allowing 96 samples to be pooled per run (and hence the cost estimates indicated above). However, the method requires an additional PCR step followed by cleaning of post-PCR products, and consequent loss of genomic material, reducing the final amount of input cDNA. On average, the ONT platform requires more input cDNA per sample than Illumina platform (1000 ng vs 0.2 ng). An emerging alternative is direct viral (RNA) sequencing which is now possible with nanopore sequencing [19]. However, this requires even larger amounts of input RNA, which may be difficult to achieve without reverse transcription and PCR. Furthermore, it is difficult to barcode native RNA (unless converted to cDNA with a barcode incorporated primer) and multiplex for cost effectiveness which is a significant residual challenge for this approach.

The ability to sequence whole RNA viral genomes with nanopore technology provides the exciting prospect of characterisation of within-host viral variants, without the need for haplotype reconstruction for the first time. As shown by us and others, using short reads to reconstruct viral haplotypes has poor consistency across the different bioinformatic algorithms used for this purpose [6, 20]. Furthermore, the uncertainty increases with the length of reconstructed haplotypes above 1.5Kb, which makes it essentially impossible to accurately reconstruct variants for the entire 9.5 kb HCV haplotype [15]. The plasmid mix and match experiments described here were able to differentiate very low frequency variants, but it also required alignment against the autologous reference sequence which is unlikely to be available when sequencing real-world clinical samples. Based on the errors observed between reads within an alignment per plasmid, the within alignment diversity due to errors of nanopore sequencing was estimated. This in turn was useful to calibrate the Hamming distance cut-off to identify between host and within host variants with the Nano-Q tool. This will be useful to researchers using the nanopore technology to sequence HCV and similar RNA viruses.

These methods have a few imitations. Viral amplicon generation is subject to technical errors generated during reverse transcription and during PCR amplification which can be minimized to an extent by using high fidelity polymerases. As mentioned above, nanopore sequencing itself has a high error rate, especially in homo-polymeric regions. This may be partially compensated for by generating a de novo viral consensus sequence and by ensuring the depth of coverage is > 300 reads per nucleotide position across the genome. The Nano-Q tool is specifically designed to analyse HCV nanopore sequences, as the pipeline makes key assumptions that are more likely to be true in HCV, but not in other viruses (for example stop codons and indels are not present within the open reading frame of functional HCV genomes). Accordingly, any observations to the contrary were considered as errors and corrected to the reference sequence. This tool cannot therefore be used in its current form for viruses known to commonly have large deletions in the genome such as HBV [21], and those that have premature stop codons in the open reading frame. However, the pipeline can be adjusted by modifying the source code, which is publicly available. For flaviviruses, such as the dengue virus, the current version of the tool can be used without modifications (data not shown).

## Conclusions

Nanopore sequencing generates HCV consensus sequences with comparable accuracy to paired-end short read sequencing technology despite a higher error rate in base calling, if appropriately compensated by coverage. Nanopore sequencing is more cost effective for high throughput sequencing than Illumina sequencing when compared under similar circumstances. However, the total yield of reads per sample depend on the amount of input DNA which is currently greater for nanopore than for Illumina sequencing. Nanopore sequencing can differentiate variants at frequencies as low as 0.1% depending on the total depth of coverage per sample. The Nano-Q tool reported here may be a useful alternative to identify full-genome length within-host variants without haplotype reconstruction.

## Methods

### Sample preparation and nanopore sequencing

The clinical samples from which genomes were extracted were sourced from the Hepatitis C Incidence and Transmission Studies in prisons and in the community (HITS-p and HITS-c) which recruited HCV seronegative and RNA negative people who inject drugs in New South Wales, Australia between 2005 and 2014. The details of this cohort are published elsewhere [22, 23] and de-identified and stored plasma / serum samples from these cohorts were used for RNA extraction. Full-length HCV amplicons were generated by reverse transcription and a nested PCR was conducted as described previously by the authors [14]. In brief, 280 μl of patient plasma was used to extract viral RNA with the QIAmp Viral RNA mini kit according to the manufacturers’ instructions (Qiagen, Chadstone Centre, Vic, Australia, Catalogue number: 52906). Reverse transcription of viral RNA was carried out using SuperScript III First-Strand Synthesis System (Catalogue number: 180851, Life Technologies, Australia) and a pan-genotype primer (oligo dA20). The nested PCR was completed with a combination of genotype specific and universal HCV primers using Takara LA taq DNA polymerase (Catalogue number: RR002B, Scientifix life, Australia) [14]. Prior to sequencing, the final products were size selected with Monarch DNA Gel Extraction Kit (catalogue number: T1020S, New England Biolabs, USA) to pick out the band of interest at approximately 10 kb by gel extraction on an 0.8% agarose gel, and subsequently purified with magnetic beads (Agencourt AMPure XP, Beckman Coulter, USA, Cat: A63881) according to the manufacturer’s instructions. Nanopore sequencing was carried out according to the manufacturer’s protocols at the Kinghorn Centre for Clinical Genomics (a licenced ONT service provider), Garvan Institute of Medical Research in Sydney, Australia with an ONT MinION or GridION sequencer on a FLO-MIN107 v9.5 flow cell. Barcoding of PCR products was performed using one of two methods: ligation barcoding where oligonucleotide barcodes were attached to the nested PCR product via ligation (ONT ligation sequencing kit SQK-LSK109); and PCR barcoding where adapters were incorporated into the PCR product during the final round of nested PCR, which in turn was used to attach a barcode by an additional PCR. In the case of PCR barcoding, the products were size selected and purified for a second time prior to sequencing. Following sequencing, the signal level data were de-multiplexed with Guppy (version 2.3.5 and 3.0.3, http://staff.aist.go.jp/yutaka.ueno/guppy/) and further processed with the Nanopolish algorithm (version 0.11.1, https://github.com/jts/nanopolish). The de-multiplexed, cleaned reads were then used for further analysis.

### Accuracy of nanopore reads in generating HCV consensus sequences

Full genome amplicons generated from the sera of four HCV-infected patients (subtype 1a) were sequenced with Nanopore technology as described above with ligation barcoding. The de-multiplexed data were aligned using GraphMap aligner (v0.5.2) [24] or Minimap2 (2.14-r921-dirty) aligner [25]. In addition, these subjects had the full genome of the virus sequenced on the Illumina MiSeq platform, as previously described [12]. A subtype-specific reference sequence was used to align the Illumina reads with Geneious Prime version 2020.0.5 (https://www.geneious.com) using the ‘map to reference’ option, with the Geneious aligner (settings: medium sensitivity / fast, 5 iterations). Two rounds of alignment were completed. The consensus from one round was used as the reference for the next round to minimize the bias from the non-autologous reference. The consensus generated from nanopore reads were compared against the Illumina-generated consensus quantitatively by counting pairwise differences. To determine the minimum coverage required for an accurate consensus sequence, different numbers of reads in multiples of 100 were randomly picked from each de-multiplexed sequence set for each patient and were aligned using a non-autologous subtype specific reference. This was compared to the nanopore consensus generated from all sequences and the pairwise differences were plotted and averaged across the four subjects.

### Sensitivity of nanopore sequencing to recover variants in a mix of sequences

Estimation of the sensitivity of nanopore sequencing to recognize low frequency variants in a relatively homogenous sequence mix was accomplished by two simulation experiments in which six HCV Envelope sequence mixtures (E1E2, length: 1800 nt. as inserts of a plasmid which was subsequently cloned and extracted) of the same subtype (1a or 1b) were combined in varied proportions to generate 15 different sample mixtures. The protocol for this step is provided in Supplementary files 1 and 2. Each HCV insert in a plasmid originated from a different patient, other than two obtained at well-separated timepoints from the same patient. The proportions of each of the 6 plasmids in a mixture varied between 0.1–93% across the 15 mixes with a uniform representation across the spectrum of prevalence. All sample mixtures were nanopore sequenced with ligation barcoding, and the de-multiplexed read outputs were aligned against each of the six reference sequences. The read count for each alignment was considered as a proxy measure of prevalence of the variant in the mixture. The consensus sequence generated per plasmid alignment was compared to the original plasmid sequences to quantify any pairwise differences. The diversity within such an alignment (attributable to technical errors of nanopore reads) was quantified and averaged across all alignments to identify the cut-off for differentiation of technical errors of nanopore sequencing from low prevalence single nucleotide polymorphisms (SNPs).

### Scale up of ONT sequencing to assess cost effectiveness

Fifty-two HCV amplicon mixes, each from a different patient sample were PCR-barcoded and pooled in a single nanopore sequencing run on a GridION platform. For this step, DNA amplicons were generated from preserved RNA from a HCV sequencing study published previously [12]. The reads were then de-multiplexed as described above and consensus sequences were generated to compare with Illumina consensus sequences for the same subjects, provided the minimum required coverage per position was met (> 300 per nt position). The cost of nanopore sequencing (plus library preparation and service charges) per patient sample were compared with that of Illumina sequencing (sequencing on a MiSeq platform with Nextera XT barcodes per sample).

### Designing a novel bioinformatic pipeline to differentiate within-host variants

The newly designed bioinformatic tool, referred to as nanopore quasi-species tool (Nano-Q) performed the following steps serially: firstly, the pipeline arranged reads according to length and then selected all reads above a user defined cut-off length; secondly, each read was cleaned using the consensus sequence as a guide; and finally, a hierarchical clustering algorithm was used to identify within-host variants. The Nano-Q tool has been implemented in Python (3.7.2) using packages implemented in Biopython [26], Scipy [27, 28], Numpy [29], Matplotlib [30], Pysam (https://github.com/pysam-developers/pysam) and is available for download at https://github.com/PrestonLeung/ONT-Tool. This fully automated pipeline has several user-defined variables which allows a conservative or a liberal approach to characterizing within host variants. In the latter approach more low abundance variants are identified but some of these may be spurious variants given the error rate of nanopore sequences. A brief summary of the bioinformatic workflow is given below. An example of an output from the Nano-Q tool is given in supplementary files 3, 4, 5, 6.

#### Pre-preparation

The de-multiplexed fastq files for each subject were first aligned to a subtype specific generic HCV reference sequence using Minimap2 [31] with parameters *-ax map-ont --MD* to produce a Sequence Alignment Map (sam file). Using *samtools view*, (version 0.1.19) this was converted to binary format (bam file) with parameter *-bSF 2304*, filtering out any secondary and supplementary alignments. The bam file was subsequently sorted and indexed using *samtools sort* and *samtools index* options. The final bam file was used as the input for Nano-Q.

#### Nano-Q tool

The Nano-Q tool adopts a conservative approach to clean nanopore reads if they meet a user-defined minimum length. Shorter reads are discarded. Using the consensus sequence as a guide, Nano-Q identified and converted base mismatches of each nanopore read to that of the consensus sequence if the quality score was below a user defined cut-off. Those above the quality score cut-off were retained as a true SNP. Similarly, as the HCV open reading frame (ORF) is translated as a single polyprotein without intervening stop codons, any indels and stop codons present in the reads were considered as technical errors and removed. In the case of stop codons, the violating codon was replaced by that of the consensus. A pairwise calculation of Hamming distances was then performed between every cleaned read [N(N-1))/2 total calculations where N is the number of reads]. These values were stored into a distance matrix and hierarchical clustering (implemented in Scipy package 1.2.1 [27, 28]) was performed to search for clusters of reads within a user defined Hamming distance threshold. This threshold was calibrated by the readouts from the HCV plasmid mixture experiments. For plasmids with an actual 5–15% pairwise difference (equivalent to between-host HCV variants), a Hamming distance cut-off of 160–180 accurately separated them into clusters where the consensus of each cluster was a near-accurate reconstruction of each plasmid. For the pair of plasmids originating from the same patient (i.e. true within-host variants with less than 5% actual pairwise difference) a Hamming distance cut off of 80–96 allowed adequate resolution to separate these clusters. A higher cut off merged these populations as a single cluster. Each plasmid has an HCV Envelope insert of approximately 1800nts; hence by linear extrapolation it is recommended to use a Hamming distance of 800–900 to differentiate between within-subject full-length variants (from a non-barcoded mix of reads originating from different subjects) and 400–480 to differentiate within-subject full-length variants (from barcoded and demultiplexed reads originating from a single host). In the next step the clusters were filtered by their size to select the largest clusters. As the final step, for each cluster, a consensus sequence was generated (a within-host variant) and these were merged if identical.

Using a lower Hamming distance threshold resulted in more read clusters, but each with a fewer number of reads. Given the high error rate in nanopore reads (up 10% compared to < 1% with Illumina technology) having many clusters with a only very few reads increased the risk of generating spurious variants. Therefore, the tool allows the user to define a minimum cluster size and those with fewer reads to be discarded. This approach allowed filtering of inappropriately corrected reads in the above steps, as these are unlikely to have a similar partner to cluster with. The number of reads within each “accepted” cluster was normalised against the total number of reads from all accepted clusters to estimate the frequency of occurrence of the viral variant within the sample. The final output of the algorithm was a sequence file in FASTA format containing all consensus sequences of variants generated from clusters, and the header of each sequence indicated the relative abundance of that variant as a fraction between 0 and 1.

Nano-Q is currently executable on Linux systems (Desktop: Ubuntu 14.04, 32 GB RAM, and an Intel Core i7–4890 3.60 Ghz, Internal Server: RedHat 6.9, 529 GB RAM and 4 × Intel Core Xeon E5-4650L 2.60) on command prompt with nine user-defined variables and three optional parameters (full parameter description and instructions available at https://github.com/PrestonLeung/ONT-Tool).

An example of the command line is shown below;

**python indelRemover003H.py -b ../example.sorted.bam -l 8500 -nr 1 -q 5 -j 50 -c 1 -ht 400 -mc 30.**

-l: length cut-off, selecting a shorter cut-off will increase the number of eligible reads and the computational requirements will also increase exponentially.

-nr: number of references for the alignment (usually one).

-q: threshold for base quality score for cleaning reads. Any base mismatch below this threshold will be considered an error and corrected to that of the consensus while those with a quality score above this value will be retained as a true SNP.

-c: starting codon (in the reference) for eligible reads. Any read without this position will be discarded. It is recommended to visually inspect the .bam file to see the first codon common to a majority of reads and select this position (number according to the reference) to make maximum use of all reads.

-ht: Hamming distance cut-off where all reads within this value will fall into a single cluster. We recommend using 400–480 to differentiate within host full-length HCV variants based on the in silico clonal experiments. Using a smaller threshold will increase the number of clusters (each with fewer reads) while larger values will reduce the number of clusters and hence the resolution to separate low frequency variants.

-mc: minimum acceptable number of reads per cluster. Only clusters with a read count above this threshold will be retained as a true cluster to generate a consensus sequence (a within host variant). If this number is lowered the number of clusters will increase and so would the estimated within host variants. However, as each cluster would have fewer reads the risk of generating spurious variants is higher as each nanopore read has a 10–15% error rate.

-l, −ht and -mc parameters significantly influence the number of low frequency variants detected. Having a larger number of reads (filtered by a shorter length) will increase the accuracy of detecting true low frequency variants. Lowering -mc or -ht will increase the number of low frequency variants detected but will have a minimum impact on the frequency of major variants if the total number of reads are high (e.g. in a dataset where the total number of eligible reads are 10,000 and a major variant is present as a cluster of 3000 reads, if -mc is set at 30, the lowest possible frequency of a minor variant will have an abundance of 0.3%. If this threshold is lowered to 20, one or more variants will appear each with an abundance of 0.2–0.3%. This is unlikely to have a significant impact on the abundance estimate of the major variant.

## Supplementary Information

**Additional file 1: Supplementary file 1.** Methods for plasmid mix experiments.

**Additional file 2: Supplementary file 2.** Sequences of HCV inserts in plasmid experiments.

**Additional file 3: Supplementary file 3–6.** An example of Nano-Q tool output (4 files in *.txt format): a) Reference sequence (HITP300157_Reference), b) Nano-Q tool progress report (HITP300157_Nano-Q_output), c) All variants generated (HITP300157_all_variants), d) Consensus of clusters of variants that met the user defined cut-off for a minimum cluster size and their relative frequencies – The final output of the tool (HITP300157_final_variants).

**Additional file 4: Supplementary file 7.** GenBank accession numbers of consensus sequences shown in Fig. 4.

**Additional file 5: Supplementary file 8**. Within-host variants generated using Nano-Q tool for subjects in Fig. 4.

## Data Availability

Nano-Q is publicly available via the Github repository at https://github.com/PrestonLeung/Nano-Q. HCV insert sequences for plasmid experiments are provided in supplementary file 2. The Illumina consensus sequences of the samples described in this manuscript have been previously uploaded to Genbank (Supplementary file 7) [12]. The within-host variant sequences generated by Nano-Q tool are provided in supplementary file 8.

## Abbreviations

AUD: Australian dollar
DNA: Deoxy-ribo nucleic acid
HBV: Hepatitis B virus
HCV: Hepatitis C virus
kb: Kilobases
NGS: Next generation sequencing
NHMRC: National health and medical research council
ONT: Oxford nanopore technology
Nano-Q: Nanopore quasispecies
RNA: Ribo-nucleic acid
SD: Standard deviation
SNPs: Single nucleotide polymorphisms
UNSW: University of New South Wales
