# Supplementary material for: Adaptation of Oxford Nanopore technology for hepatitis C whole genome sequencing and identification of within-host viral variants
Source: BMC Genomics. 2021 Mar 2;22:148. doi: 10.1186/s12864-021-07460-1 (PMC7923462; doi:10.1186/s12864-021-07460-1)
Supplement: Supplementary file 1 — Additional file 1: Supplementary file 1. Methods for plasmid mix experiments. [file 12864_2021_7460_MOESM1_ESM.docx]

**Plasmid purification and DNA quantification:**

A total of 10 plasmids containing HCV E1E2 insert of around 1.75kbp length were used in Mix and match experiments:

| **Serial #** | **Plasmid ID** | **Genotype** | **Vector** | **Host strain** |
| --- | --- | --- | --- | --- |
| 1 | pSOMS129 | 1a | pcDNA3.1 | DH5α |
| 2 | pSOMS132 | 1a | pcDNA3.1 | DH5α |
| 3 | pSOMS133 | 1a | pcDNA3.1 | DH5α |
| 4 | pSOMS47 | 1a | pcDNA3.1 | DH5α |
| 5 | pSOMS48 | 1a | pcDNA3.1 | DH5α |
| 6 | pSOMS50 | 1b | pcDNA3.1 | DH5α |
| 7 | pSOMS51 | 2a | pcDNA3.1 | DH5α |
| 8 | pSOMS56 | 3a | pcDNA3.1 | DH5α |
| 9 | pSOMS58 | 4 | pcDNA3.1 | DH5α |
| 10 | pSOMS61 | 6 | pcDNA3.1 | DH5α |

Plasmids were purified from bacterial cultures of 200 mL, grown overnight in Luria-Bertani (LB) broth with 50 μg/mL ampicillin. Extraction was performed using Nucleobond Xtra midi kit (Scientifix, Cat no: 740410) as per manufacturer’s instructions with the exception that plasmids were reconstituted in nuclease-free water (Ambion, Thermo Fisher, Cat no.4387936). DNA Quantification of undiluted DNA was performed using a NanoDrop ND-1000 UV spectrophotometer (Thermo Scientific). Absorbance at 260 nm and 280 nm were measured to determine DNA concentration and purity.

**PCR Amplification of Plasmids**

A 50μL reaction consisting of following components and concentrations was prepared for the PCR amplification of plasmids:

| **Component** | **50 ul reaction** | **Final concentration** |
| --- | --- | --- |
| Nuclease free water | to 50ul |  |
| 10x PCR buffer, -Mg | 5ul | 1x |
| 50mM MgCl2 | 1.5ul | 1.5mM |
| 10 mM dNTP mix | 1ul | 0.2mMeach |
| Platinum Taq DNA polymerase | 0.2ul | 2 U/rxn |
| Primer T7 + (10uM) | 1ul | 0.2uM |
| Primer BGH - (10uM) | 1ul | 0.2uM |
| Template DNA | 7 ul |  |

PCR was carried out by performing an initial denaturation cycle at 94°C for two minutes, followed by 35 cycles at 94°C for 30 seconds, 55°C for 30 seconds and 72°C for two minutes.

Products were then run on a 1.5% agarose gel.

Primers used in PCR amplification:

T7 + TAATACGACTCACTATAGG (5'-3')

BGH – TAGAAGGCACAGTCGAGG (5'-3')

a (+) indicates that the oligonucleotide is a forward primer and a (–) indicates a reverse primer.

**DNA purification**

DNA was purified using Agencourt AMPure XP (Beckman Coulter, Cat no. A63882) system as per manufacturer’s instructions. To purify DNA, 1.8 volumes of Agencourt AMPure XPwas added to one volume of PCR product, mixed and left at room temperature for five minutes. The reaction was then applied to a magnet for two minutes to separate the beads from the solution. Supernatant was then aspirated and washed twice with 200 μL of 70% ethanol. To elute, the reaction was removed from the magnet and 40 μL of nuclease-free water (Ambion, Thermo Fisher, Cat no. 4387986) was mixed with the beads. Supernatant was collected and then used immediately or stored at -20°C.

**DNA quantification and Mix preparation**

PCR amplicons were quantified with the Quant-iT PicoGreen dsDNA assay (Invitrogen, Cat no. P7589) according to the manufacturer protocol. After the quantification, all the PCR clones from different HCV genotypes were normalized and serially diluted to different concentrations. Then, a maximum of five different clones were mixed to form a clonal mix. Ultimately, 20 different mixes were constituted with different clonal components and different concentrations.
